# Emerging Extraction Technologies and Molecular Implications for Greek Olive Products: A Commentary

**DOI:** 10.3390/molecules31172961

**Published:** 2026-08-25

**Authors:** Vassilis Athanasiadis

**Affiliations:** Department of Food Science and Nutrition, University of Thessaly, Terma N. Temponera Street, 43100 Karditsa, Greece; vaathanasiadis@uth.gr

**Keywords:** phenolics, antioxidants, olive oil quality, Greek cultivars, processing effects, olive by-products, pulsed electric field, sustainability, circular economy, quality markers

## Abstract

Recent advances in non-thermal and hybrid extraction technologies—such as pulsed electric field (PEF), ultrasound-assisted extraction (UAE), microwave-assisted extraction (MAE), and enzymatic treatments—have been widely reviewed in the context of general food processing. However, the current literature lacks a critical evaluation of how these modalities reshape the molecular fingerprints, authenticity markers, and cultivar-specific phenolic baselines of olive products, particularly within the Greek production landscape. Existing studies primarily emphasize extraction yield, operational parameters, or sustainability aspects, leaving important gaps regarding molecular consequences, including shifts in secoiridoids, lignans, pigments, oxidation markers, and the composition of by-products such as olive mill wastewater and pomace. This commentary addresses these gaps by integrating emerging extraction technologies with Greece’s omics-enabled analytical capacity (FoodOmicsGR_RI), highlighting how processing innovations may influence authenticity claims, phenolic integrity, and circular economy valorization routes. We discuss mechanistic pathways, molecular-level effects, and technology-specific limitations and propose a structured framework for developing national databases of processing-induced molecular markers. By linking technological mechanisms with cultivar-dependent molecular responses, this commentary aims to support coordinated Greek research efforts toward robust authenticity assurance, sustainable processing, and high-value valorization of olive by-products under evolving climatic and industrial pressures.

## 1. Introduction: Molecular Characterization and the Greek Research Landscape

Greece has established a mature omics-enabled research ecosystem for the molecular characterization of olive products. FoodOmicsGR_RI is a central pillar of this infrastructure, integrating NMR, LC–MS/MS, and other analytical platforms to develop cultivar- and origin-specific molecular signatures for Greek olive oils and table olives [1,2]. This national capacity offers a unique platform for studying the effect of novel extraction technologies on the molecular composition, phenolic integrity, and authenticity markers of Greek olive matrices.

Despite the extensive global literature on emerging extraction technologies such as PEF, UAE, MAE and enzymatic treatments, three major gaps remain unaddressed: (i) the lack of studies linking these modalities with omics-based molecular fingerprints relevant to authenticity and cultivar discrimination; (ii) insufficient evaluation of processing-induced molecular shifts in Greek cultivars, which exhibit distinct phenolic biosynthesis patterns and climate-responsive molecular behavior; (iii) limited understanding of how these technologies reshape the molecular composition of olive by-products within circular economy frameworks. Existing reviews usually concentrate on extraction yield, operational parameters or general sustainability aspects, without incorporating these technologies into a cohesive molecular framework pertinent to authenticity assurance, cultivar differentiation and valorization.

By connecting newly developed extraction techniques with the omics-based molecular fingerprints previously developed for Greek olive oils and table olives, the current commentary presents a novel conceptual viewpoint. This integration is more than just descriptive; it draws attention to unanswered scientific and practical issues about how processing advancements might alter important oxidation signatures, lignans, pigments, and phenolic markers that support quality and origin verification. These molecular shifts may also influence sensory attributes, including bitterness, pungency, LOX-derived volatiles and pigment-driven color, underscoring the need for integrated molecular–sensory evaluation frameworks.

Additionally, FoodOmicsGR_RI is one of the few national infrastructures worldwide capable of producing harmonized NMR/LC–MS/MS datasets across cultivars, regions and processing styles. This capacity makes Greece an exceptional case study for evaluating processing-related molecular variation. In contrast, countries such as Spain and Italy have already conducted pilot-scale trials of PEF and UAE, supported by larger industrial infrastructures and higher technology readiness. Greece’s milling sector—dominated by small and medium-sized mills—faces different economic, regulatory and infrastructural constraints, highlighting the need for Greece-specific feasibility assessments rather than direct adoption of international models.

To prevent confusing cultivar-specific signatures and to guarantee regulatory compliance with EU definitions of virgin olive oil, new extraction technologies must be assessed within standardized molecular baselines. The article suggests a strategic framework for creating national databases of processing-induced molecular markers and for directing the sustainable valorization of olive by-products by combining technological mechanisms, molecular consequences and industrial feasibility. Industrial adoption in Greece remains limited due to equipment cost, infrastructure requirements, regulatory uncertainty and the fragmented structure of the milling sector—factors that must be considered when interpreting molecular benefits.

Recent Greek studies showed that phenolic fingerprints, oxidation markers, and non-targeted metabolomic profiles can efficiently discriminate cultivars (e.g., Koroneiki and Kolovi) and different geographical origins. Metabolomics, combined with chemometric modeling, can identify molecular markers related to processing style, environmental parameters, and regional terroir, providing a solid baseline for comparison of new extraction modalities [2,3,4,5]. A further gap concerns climate-driven variability, as Greek cultivars exhibit strong year-to-year fluctuations in phenolic biosynthesis, yet few studies examine how emerging extraction technologies interact with climate-responsive molecular baselines.

There is a practical and scientific need to relate processing innovations such as pulsed electric field (PEF), ultrasound-assisted extraction (UAE), microwave-assisted extraction (MAE), and enzymatic treatments with molecular-level outcomes relevant to bioactive preservation, authenticity support, and circular economy valorization of olive by-products such as alperujo, olive mill wastewater (OMWW), and pomace in this context. Green extraction and biorefinery paradigms [6,7,8] demonstrate how environmentally friendly methods can be applied to recover high-value components from olive by-products, in line with EU sustainability directives and Greek circular economy initiatives currently in progress.

Integration of omics-based authentication and green extraction technologies provides a strategic roadmap for the development of national molecular marker databases, including processing-induced changes in key phenolics such as hydroxytyrosol (HT), tyrosol, oleuropein derivatives, oleacein, and oleocanthal, as well as lignans such as 1-acetoxypinoresinol (1-AP) and pinoresinol. The parallel characterization of by-product matrices (OMWW, pomace, and leaves) under climate-driven varietal responses further strengthens the molecular understanding of Greek olive production systems [2,9,10].

Finally, the science–policy interface is gaining more relevance: consumer education and authenticity claims [11] imply that molecular markers should be reported transparently and in an intelligible manner. For Greece, where PDO/PGI certification and consumer trust increasingly rely on molecular evidence, standardized reporting of processing-induced molecular changes is essential for regulatory alignment, traceability and communication of added value. This highlights the necessity of standardization of the impact of emerging extraction technologies on the molecular profiles of olive products, beyond the extraction yields to promote trust, traceability, and the added value of Greek olive products.

## 2. Overview of Innovative Extraction Technologies and Mechanistic Relevance

Emerging extraction technologies offer new ways to modulate the molecular composition of olive products, but their relevance cannot be understood solely through mechanistic descriptions. While PEF, UAE, MAE and enzymatic treatments have been extensively characterized in the general food processing literature, their molecular consequences for authenticity, cultivar-specific phenolic baselines and Greek production systems remain insufficiently explored. This section therefore provides a critical overview of these modalities, emphasizing not only their mechanistic principles but also their potential to reshape key phenolics, lignans, pigments and oxidation markers that underpin quality and origin verification.

In contrast to countries such as Spain and Italy—where pilot-scale trials of PEF and UAE have already been integrated into industrial lines—Greece’s milling sector is dominated by small and medium-sized mills, making large-scale adoption more challenging. As a result, the evaluation of emerging technologies must consider Greek-specific constraints, including infrastructure limitations, regulatory interpretation, and the need to maintain cultivar-specific molecular fingerprints established through FoodOmicsGR_RI.

**Figure 1:**
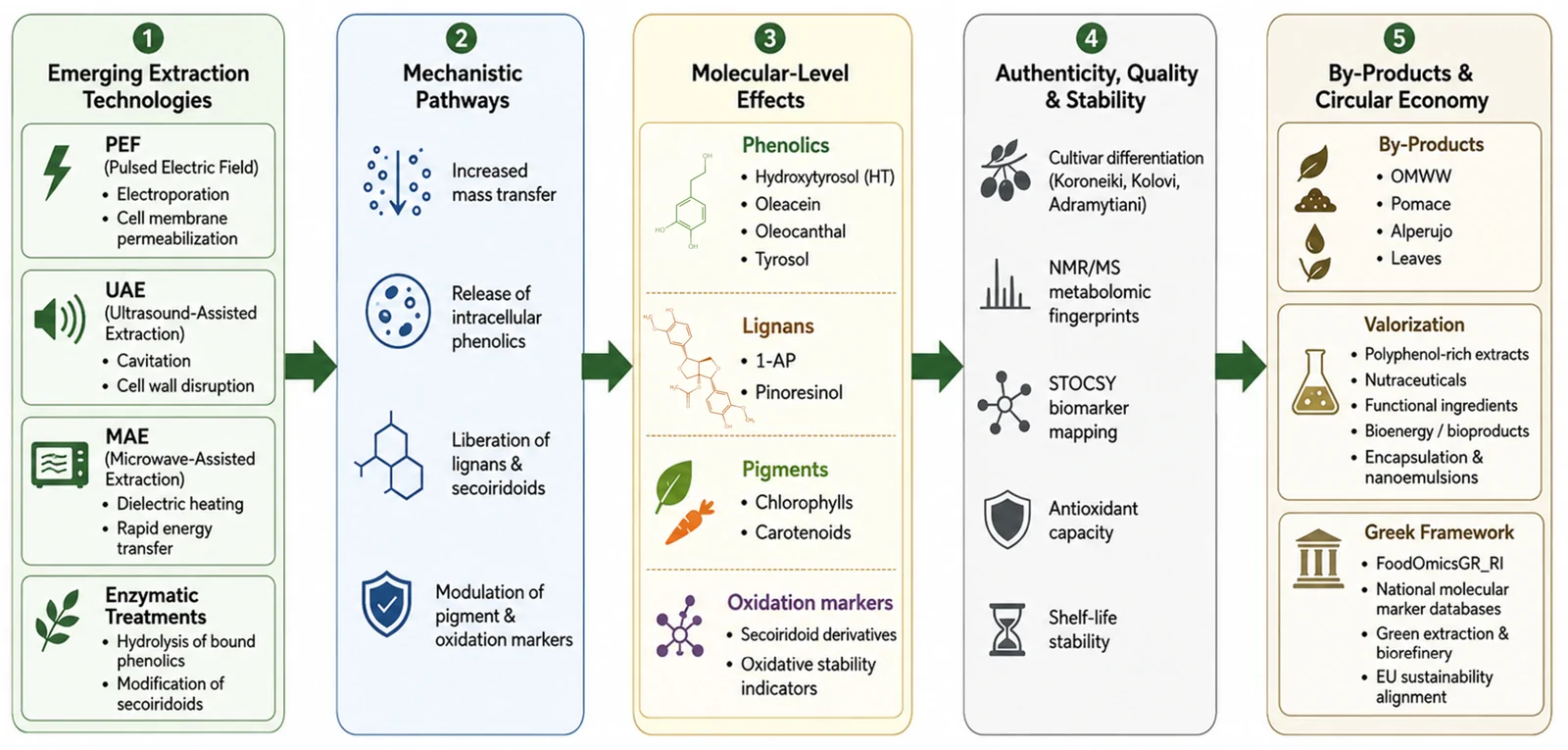
Conceptual overview of emerging extraction technologies (PEF, UAE, MAE and enzymatic treatments) and their mechanistic pathways, molecular effects, authenticity implications and circular economy valorization routes in Greek olive processing systems.

Within this context, the mechanistic pathways of electroporation, acoustic cavitation, dielectric heating and enzymatic hydrolysis are not presented here as textbook descriptions, but as drivers of molecular change with direct implications for authenticity, sensory quality and by-product valorization. These mechanisms influence the release, transformation and stability of phenolics, secoiridoids and pigments—molecules that are central to nutritional value, oxidative stability and cultivar discrimination. Figure 1 summarizes these mechanistic principles and highlights how each modality may introduce processing-specific molecular signatures relevant to Greek olive matrices and circular economy strategies.

### 2.1. Pulsed Electric Field (PEF)

Pulsed electric field (PEF) processing induces electroporation of olive mesocarp cells, enhancing mass transfer and increasing the release of phenolics, lipophilic constituents and other bioactives under minimal thermal load. This mechanistic advantage can modulate the phenolic and secoiridoid profile of olive paste and influence the composition of by-products, potentially improving extraction efficiency and the recovery of heat-labile antioxidants [12,13]. Pilot-scale studies show that PEF-treated olive paste increases the extractability of oil with no changes in sensory properties. Specific energy (kJ/kg) as an energy input parameter and phenolic retention and extraction yield are correlated. These findings are relevant for Greek cultivars such as Koroneiki and Kolovi, which exhibit strong phenolic responsiveness to cell disruption technologies [13].

Beyond yield improvements, PEF-induced phenolic enrichment has molecular implications: shifts in hydroxytyrosol, oleacein, oleocanthal and related secoiridoids may strengthen authenticity signals when monitored through NMR/MS metabolomics [14,15]. FoodOmicsGR_RI provides the analytical infrastructure needed to evaluate whether PEF-driven molecular changes remain compatible with established cultivar-specific fingerprints and do not obscure origin verification frameworks [12,13].

However, the Greek industrial context presents significant barriers to adoption. The majority of Greek olive mills are small or medium-sized, operating with limited capital and infrastructure. Installing high-voltage pulse generators requires substantial investment, dedicated safety systems and electrical upgrades that many mills cannot accommodate. Retrofitting existing malaxation lines is technically complex, and continuous PEF operation increases energy demand—an important consideration for mills already facing high operational costs.

Long-term stability data for PEF-treated oils remain limited, raising concerns about potential impacts on oxidation pathways, pigment integrity and sensory evolution during storage. These uncertainties are particularly relevant for Greek producers who rely on stable bitterness, pungency and LOX-derived volatiles as part of cultivar identity and PDO/PGI specifications.

Regulatory constraints further complicate adoption. Under EU Regulation (EU) 2568/91, PEF must be classified as a processing aid, requiring mills to demonstrate that electroporation does not alter the essential characteristics of virgin olive oil. This regulatory burden is more challenging for small Greek mills lacking technical documentation capacity. In contrast, countries such as Spain and Italy—where larger industrial plants have piloted PEF systems—have more resources to support regulatory validation and equipment integration.

Overall, while PEF offers mechanistic advantages and clear molecular potential, its feasibility in Greece depends on economic viability, regulatory clarity, and compatibility with cultivar-specific molecular baselines. These factors explain the limited industrial penetration of PEF despite promising laboratory and pilot-scale results [16,17,18,19,20].

### 2.2. Ultrasound-Assisted Extraction (UAE)

Ultrasound-assisted extraction (UAE) operates through acoustic cavitation, which disrupts cell walls and enhances solvent penetration, increasing the release of hydroxytyrosol, tyrosol and secoiridoid precursors such as oleacein and oleocanthal. These shifts can alter the relative abundances of phenolics detected by NMR- and MS-based metabolomics, making UAE particularly relevant for molecular-level monitoring [3,21,22].

UAE is widely applied in green extraction processes and enables efficient recovery of both hydrophilic and lipophilic phenolics using benign solvents. When combined with STOCSY and chemometrics, UAE-induced changes can be mapped to cultivar-specific biomarkers, supporting differentiation of processing styles and authenticity assessment [3,23]. In Greece, FoodOmicsGR_RI provides the analytical infrastructure needed to evaluate whether UAE-driven phenolic shifts remain compatible with established molecular fingerprints of cultivars such as Koroneiki and Kolovi.

However, the Greek industrial context presents important limitations. Acoustic cavitation can generate free radicals and localized hotspots, which may accelerate oxidation or degrade sensitive secoiridoids if not carefully controlled. Maintaining consistent cavitation intensity in viscous olive paste is technically challenging, and industrial-scale ultrasound systems require durable transducers and high energy input—factors that increase operational costs for Greek mills, most of which are small or medium-sized. Scale-up difficulties may lead to inconsistent phenolic profiles, complicating authenticity verification and sensory stability.

Concerns also exist regarding the impact of UAE on carotenoid and chlorophyll stability, pigments that contribute to both oxidative behavior and sensory perception. These molecular–sensory interactions are particularly relevant for Greek PDO/PGI products, where color and bitterness/pungency are part of cultivar identity. Furthermore, the introduction of high-energy acoustic fields may require additional validation to comply with EU definitions of virgin olive oil, limiting regulatory acceptance and slowing industrial adoption [17,24,25,26,27,28].

Overall, UAE offers mechanistic advantages and clear potential for phenolic enrichment, but its feasibility in Greece depends on cost, scale-up capacity, regulatory clarity and compatibility with cultivar-specific molecular baselines. These factors explain why UAE remains promising at laboratory scale yet underutilized in Greek industrial practice.

### 2.3. Microwave-Assisted Extraction (MAE)

Microwave-assisted extraction (MAE) relies on dielectric heating to accelerate mass transfer and disrupt olive mesocarp microstructure, leading to rapid extraction of phenolics and potential modulation of pigment and secoiridoid profiles. These molecular shifts can influence antioxidant capacity and cultivar discrimination, making MAE relevant for metabolomics-based monitoring of Greek olive matrices [3,22,23].

Studies combining MAE with targeted and untargeted metabolomics have shown cultivar-dependent responses, particularly in chlorophyll and carotenoid behavior—pigments that contribute to both oxidative stability and sensory perception. For Greek cultivars such as Koroneiki and Kolovi, where pigment composition is closely tied to PDO/PGI identity, MAE-induced pigment shifts must be evaluated carefully to ensure compatibility with established molecular fingerprints generated through FoodOmicsGR_RI.

Despite its mechanistic advantages, MAE faces substantial feasibility challenges in the Greek industrial context. Dielectric heating can create thermal hotspots, which may degrade volatile compounds, phenolics and pigments if not precisely controlled. This is particularly problematic for Greek mills, most of which operate small-scale malaxation lines where uniform microwave penetration is difficult to achieve due to the heterogeneous water–oil matrix of olive paste. Unlike botanical extractions—where MAE is widely adopted—olive paste presents viscosity and phase-distribution constraints that reduce heating uniformity and increase the risk of quality deterioration.

Industrial-scale microwave systems require high-power electromagnetic units, shielding infrastructure and strict safety protocols, all of which impose significant capital and operational costs. These requirements are difficult to meet for the majority of Greek mills, which are small or medium-sized and lack the space, electrical capacity and economic flexibility to integrate MAE into continuous processing lines. In contrast, countries such as Spain and Italy have explored MAE in controlled pilot environments, supported by larger industrial facilities and dedicated R&D units.

Regulatory considerations further limit adoption. Under EU Regulation (EU) 2568/91, MAE must be classified as a processing aid, requiring mills to demonstrate that microwave exposure does not alter the essential characteristics of virgin olive oil. This regulatory burden is particularly challenging for Greek producers who rely on stable sensory attributes—bitterness, pungency and LOX-derived volatiles—as part of cultivar identity and authenticity claims.

Overall, while MAE offers rapid extraction kinetics and potential phenolic enrichment, its industrial viability in Greece remains constrained by cost, safety requirements, heating uniformity challenges and regulatory uncertainty. These factors explain why MAE remains promising at laboratory scale but has not yet achieved meaningful penetration in Greek olive oil production systems.

### 2.4. Enzymatic Treatments

Enzymatic treatments aim to hydrolyze cell-wall polysaccharides and release bound phenolics, increasing the recovery of hydroxytyrosol, tyrosol and secoiridoid derivatives. These molecular shifts can enrich the phenolic fraction of both olive oil and by-products such as OMWW and pomace, offering potential advantages for nutraceutical-grade extracts and valorization streams [21,22,29]. When monitored through NMR/MS metabolomics, enzyme-induced changes can be mapped to cultivar-specific fingerprints, supporting authenticity assessment and processing differentiation.

However, the Greek industrial context presents significant constraints. The effectiveness of commercial enzyme blends varies widely across cultivars, maturity stages and paste composition. Greek cultivars such as Koroneiki and Kolovi exhibit distinct cell-wall architectures and secoiridoid profiles, meaning that enzyme performance is not easily predictable and often requires cultivar-specific optimization. This variability complicates adoption for Greek mills, which typically operate with limited technical support and cannot afford extensive trial-and-error optimization.

Operationally, enzymatic treatments introduce additional complexity: precise control of temperature, pH and reaction time is required to avoid over-hydrolysis, which can destabilize emulsions during malaxation or negatively affect sensory attributes such as bitterness, pungency and LOX-derived volatiles. These sensory implications are particularly relevant for Greek PDO/PGI products, where cultivar-specific sensory profiles are integral to authenticity claims.

Cost is another major barrier. Enzymes represent recurring expenses rather than one-time investments, making them less attractive for small and medium-sized Greek mills that operate on narrow profit margins. In contrast, larger industrial plants in Spain and Italy have conducted pilot-scale trials with enzymatic malaxation, supported by higher processing volumes and more flexible economic structures.

Regulatory constraints further limit feasibility. Under EU Regulation (EU) 2568/91, exogenous enzymes must be classified as processing aids, requiring mills to demonstrate that enzymatic hydrolysis does not alter the essential characteristics of virgin olive oil. This regulatory burden is particularly challenging for Greek producers, who often lack the documentation capacity and analytical infrastructure needed for compliance. FoodOmicsGR_RI can support this process by providing harmonized metabolomics workflows to evaluate whether enzyme-induced molecular shifts remain compatible with established Greek cultivar fingerprints.

Finally, enzymatic treatments can alter the molecular profile of by-products in unpredictable ways, affecting oxidation markers, lignan stability and phenolic composition. These changes complicate valorization strategies, especially for applications requiring consistent molecular profiles (e.g., nutraceutical extracts, functional ingredients). As a result, despite promising laboratory results, enzymatic malaxation has achieved only marginal industrial penetration in Greece, constrained by cost, variability, regulatory uncertainty and the need to maintain authenticity and sensory stability [30,31,32,33].

### 2.5. Cross-Cutting Implications and Greek Context

Across all four modalities—PEF, UAE, MAE and enzymatic treatments—emerging extraction technologies introduce processing-driven molecular shifts that must be interpreted within the established Greek omics baselines. These technologies can alter phenolics, pigments, lignans and oxidation markers in ways that directly affect authenticity verification, sensory stability and the valorization potential of olive by-products [2,7,8]. For Greece, where cultivar identity (e.g., Koroneiki, Kolovi, Adramytiani) is tightly linked to phenolic ratios, LOX-derived volatiles and pigment composition, even small processing-induced molecular changes can influence PDO/PGI specifications and consumer expectations.

Integrated metabolomics is therefore essential for capturing molecule-level changes in EVOO phenolics, pigments and oxidation markers. FoodOmicsGR_RI provides harmonized NMR/LC–MS/MS workflows that allow Greek researchers to distinguish between cultivar-intrinsic variation and technology-induced molecular shifts. This capability is particularly important because Greek cultivars exhibit strong climate-driven variability, making it necessary to evaluate whether emerging technologies amplify, mask or distort climate-responsive molecular signatures.

Parallel profiling of by-products (OMWW, alperujo, leaves and pomace) is equally critical. Emerging technologies can increase phenolic liberation or modify lignan and secoiridoid stability, reshaping the molecular pools available for valorization. In Greece, where circular economy initiatives are expanding but industrial infrastructure remains fragmented, understanding these molecular shifts is key to designing realistic biorefinery pathways—from nutraceutical extracts to functional ingredients and antioxidant-rich formulations.

The Greek industrial landscape also shapes cross-cutting implications. Most Greek mills are small or medium-sized, operating with limited capital, heterogeneous equipment and variable processing conditions. As a result, the molecular effects of emerging technologies may differ substantially from those observed in large industrial plants in Spain or Italy. Ensuring reproducibility across Greek mills requires standardized protocols, shared analytical baselines and coordinated pilot-plant trials that reflect real processing conditions rather than idealized laboratory setups.

Overall, the integration of omics-based authentication with emerging extraction technologies offers Greece a strategic advantage—but only if molecular shifts are systematically mapped, interpreted within cultivar-specific baselines and aligned with regulatory and industrial realities. This Greek-specific synthesis is essential for ensuring that technological innovation strengthens authenticity, supports sensory stability and enables sustainable valorization rather than introducing new sources of variability or regulatory uncertainty.

### 2.6. Industrial Feasibility, Regulatory Constraints and Technology Readiness

Although emerging extraction technologies such as PEF, UAE, MAE and enzymatic treatments offer clear mechanistic advantages, their industrial adoption in Greece remains limited due to economic, infrastructural and regulatory constraints. Most Greek olive mills are small or medium-sized, making the integration of high-voltage PEF systems [34], high-power ultrasound units [16,17,18,24,25], or microwave reactors [35,36] financially and technically challenging. Enzymatic treatments introduce recurring costs and require precise control of temperature, pH and reaction time, which is difficult to maintain under heterogeneous Greek milling conditions [30,31,32,33].

Technology readiness levels (TRLs) for these modalities remain moderate. PEF has reached pilot-plant testing in European mills [34], but full industrial integration is rare due to equipment cost, retrofitting complexity and energy requirements [36,37]. UAE and MAE, although established in botanical extraction industries [16,17,18], face scale-up limitations in olive paste processing because of viscosity, phase heterogeneity and oxidative stability concerns [38]. Enzymatic malaxation has been tested industrially [30,33], but cultivar-dependent variability and sensory risks limit widespread use [19,31].

Regulatory constraints further slow adoption. Under Regulation (EU) 2568/91, all modalities involving external energy fields or exogenous enzymes must be classified as processing aids, requiring mills to demonstrate that the essential characteristics of virgin olive oil remain unchanged [19,30,39,40,41,42]. This documentation burden is particularly challenging for Greek producers, who often lack the analytical infrastructure needed for compliance.

Cost-related barriers are also significant. PEF systems require high-voltage pulse generators and dedicated safety infrastructure [16,17,19,20,41,43]. UAE may generate free radicals and localized heating, raising concerns about oxidative stability and phenolic degradation [16,19,20]. MAE can create thermal hotspots that degrade pigments and volatiles while also requiring shielding and high-power electromagnetic units [35,36]. Enzymatic treatments involve recurring expenses and exhibit variable performance depending on cultivar and maturity stage [30,31,32,33].

Patent activity reflects limited industrial interest: few olive-specific patents exist for PEF, UAE or MAE, and enzymatic malaxation has not produced widely adopted commercial solutions [16,17,19,20]. As a result, conventional malaxation remains dominant due to its regulatory clarity, robustness and predictable sensory outcomes.

Overall, the industrial viability of emerging extraction technologies in Greece is shaped more by cost, infrastructure, regulatory uncertainty and cultivar reproducibility than by scientific limitations. Any interpretation of molecular benefits must therefore consider realistic processing conditions and the structural characteristics of the Greek olive oil sector.

## 3. Impact on Bioactive and Molecular Composition

The molecular composition of olive products is affected by new extraction technologies by modulating the release, stability and transformation of major phenolics, lignans, pigments and markers of oxidation. These molecular modifications directly affect nutritional quality and antioxidant capacity. They also influence origin verification and cultivar discrimination. The molecular effects are summarized below along two integrated thematic axes: (i) key molecular classes affected by processing and (ii) implications for authenticity and cultivar-specific fingerprints.

### 3.1. Key Molecular Classes Affected by Emerging Technologies

Hydroxytyrosol (HT), oleacein and oleocanthal remain central discrimination features for cultivar, origin and quality [2,3,4,5]. Their concentrations are highly responsive to cell disruption, making them particularly sensitive to modalities such as PEF, UAE, MAE and enzymatic treatments. Because these secoiridoids contribute to bitterness, pungency and oxidative stability, processing-induced shifts must be monitored to ensure that technological interventions do not distort cultivar-specific sensory profiles or PDO/PGI specifications. NMR/MS-based metabolomics provides the resolution needed to track these changes and distinguish technology-driven variation from natural phenolic variability.

Lignans such as 1-acetoxypinoresinol (1-AP) and pinoresinol are influenced by cultivar, maturity and extraction conditions. Emerging technologies may alter lignan retention or transformation, with implications for nutritional value and authenticity [10]. For Greek cultivars, where lignan ratios contribute to molecular fingerprinting, understanding lignan behavior under different extraction regimes is essential for maintaining consistency with established omics baselines.

Phenolics and pigments jointly determine antioxidant capacity and oxidative stability. Greek metabolomics studies have shown strong correlations between chlorophyll/carotenoid profiles and oxidative markers [3,4,5,12]. Technologies that enrich phenolics or modulate pigment composition may improve oxidative stability, but they may also introduce variability in color and LOX-derived volatiles, requiring careful molecular–sensory monitoring.

### 3.2. Authenticity, Fingerprints and Processing-Induced Variability

NMR- and MS-based metabolomics provide high-resolution metabolic profiles capable of distinguishing Greek cultivars and geographical origins. Emerging extraction technologies introduce processing-driven molecular variations that must be mapped against these established fingerprints to prevent confounding origin signals. Tools such as STOCSY can help differentiate processing effects from cultivar-intrinsic variation [2,4,5], ensuring that authenticity markers remain interpretable even when new technologies are applied.

The FoodOmicsGR_RI infrastructure offers harmonized molecular datasets for Greek EVOOs and olives, enabling researchers to interpret processing effects within climate-responsive and cultivar-specific baselines [2,3,13]. This is particularly important for Greek cultivars, which exhibit strong year-to-year phenolic variability driven by climate conditions. Integrating processing studies with national omics datasets strengthens the ability to evaluate whether emerging technologies enhance or distort authenticity markers, sensory attributes and oxidation signatures.

Processing also affects the molecular composition of by-products such as OMWW and pomace. These matrices exhibit technology-dependent phenolic and lignan profiles, which determine their suitability for valorization routes aligned with EU sustainability goals [6,7,9,10,12]. Understanding how emerging technologies reshape these molecular pools is essential for designing realistic biorefinery strategies in Greece, where industrial infrastructure is fragmented and valorization pathways must be adapted to small-scale production systems.

**Figure 2:**
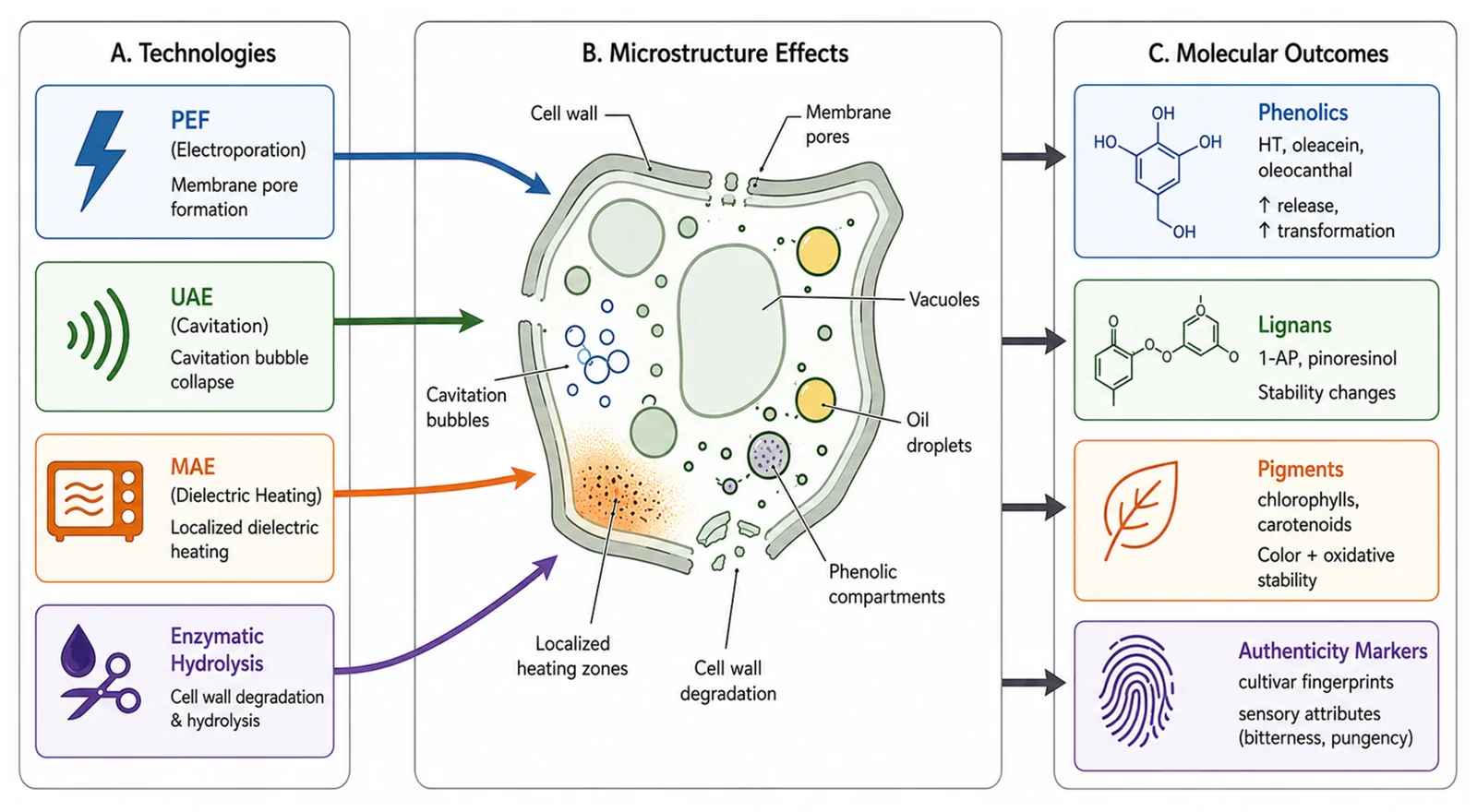
Mechanistic overview of how emerging extraction technologies (PEF, UAE, MAE, enzymatic treatments) interact with olive paste microstructure to modulate phenolic release, lignan stability, pigment composition and authenticity-relevant molecular markers. The figure summarizes the main pathways through which cell disruption and microstructural changes reshape key molecular classes that influence sensory quality, oxidative stability and cultivar-specific fingerprints.

Figure 2 provides a mechanistic and molecular overview of how emerging extraction technologies interact with olive paste microstructure and reshape phenolic, lignan, pigment and origin-relevant molecular indicators.

## 4. Implications for Olive By-Products and Circular Economy

Emerging extraction technologies reshape the molecular composition of olive by-products, influencing their valorization potential within circular economy frameworks. In Greece—where most mills are small or medium-sized and by-product management remains fragmented—understanding these molecular shifts is essential for designing realistic, scalable and economically viable biorefinery pathways. The following subsections synthesize the key molecular, technological and sustainability implications.

### 4.1. Composition and Challenges of Olive By-Products

In olive mill wastewater (OMWW), pomace and other residual streams, hydroxytyrosol, tyrosol, caffeic acid, *p*-coumaric acid and related polyphenols are present in large amounts. Phenolic pools with antioxidant potential are also abundant in olive leaves and pruning residues [7,8,44]. However, their distribution, stability and extractability depend heavily on cultivar, maturity and processing conditions.

Greek by-products present additional challenges: high organic load, phenolic complexity, seasonal variability and matrix heterogeneity. These factors complicate extraction efficiency and require careful selection of processing strategies to maximize recovery while maintaining molecular stability.

### 4.2. Effects of Innovative Extraction Technologies on By-Product Molecular Profiles

The molecular profile of by-products can be dramatically affected by emerging extraction technologies. The application of PEF, UAE and MAE can lead to an increase in phenolic liberation into OMWW and pomace through the increase in cell disruption and mass transfer, while enzymatic treatments can cause the release of bound phenolics and thus an increase in the concentration of recoverable bioactives in residual streams [7,8,12]. These molecular shifts can be advantageous for valorization, but they also introduce variability in oxidation markers, lignan stability and pigment composition.

For Greece, where valorization pipelines must adapt to small-scale production systems, profiling by-products under each technology is essential. NMR/MS-based metabolomics can identify processing-induced changes in phenolic stability, lignan content and oxidation signatures, determining suitability for nutraceutical, cosmetic or food-grade applications.

### 4.3. Valorization Opportunities

The increased phenolic content in OMWW, pomace and leaf residues offers a number of valorization opportunities. Recovered polyphenols can be used as nutraceutical ingredients, functional food additives or natural antioxidants in active packaging systems. Other opportunities are bioenergy production, biopolymers and bioproducts from OMWW and pomace. Encapsulation and nanoemulsion technologies are capable of stabilizing recovered phenolics, improving bioavailability, and enabling their incorporation into high-value formulations [7,8,45,46].

However, economic feasibility in Greece remains constrained by small production volumes, limited infrastructure, and the need for standardized extraction protocols. Valorization routes must therefore be tailored to regional capacities rather than assuming large-scale industrial biorefineries.

### 4.4. Alignment with EU Sustainability Goals and Greek Circular Economy Initiatives

Emerging extraction strategies are consistent with EU priorities in green chemistry, biorefinery development and resource efficiency. In Greece, national initiatives like FoodOmicsGR_RI and the regional “olive road” programs provide a framework to test integrated valorization pipelines where molecular profiling is coupled with sustainable extraction technologies [2,7,8]. These initiatives enable the testing of valorization pipelines where omics-based characterization is directly linked to environmentally responsible processing approaches, ensuring that recovered phenolics and lignans meet quality, safety and authenticity requirements.

Standardized metabolomics workflows among Greek institutions may support the creation of national databases of processing-induced molecular markers enabling traceability, authenticity assurance and sustainability claims for both olive oils and derived by-products. Such databases enhance traceability, authenticity assurance and evidence-based sustainability claims, aligning Greek production systems with EU circular economy directives. By coupling molecular insights with realistic valorization strategies adapted to the scale and structure of Greek olive mills, these efforts strengthen Greece’s position in the transition toward a circular bioeconomy without overstating industrial feasibility.

## 5. Future Directions for Greek Research

The advancement of the molecular characterization of Greek olive products requires harmonization of analytical workflows at national level, climate-responsive monitoring, cross-institutional collaboration and coordinated methodological innovation. Future research should focus on integrating emerging extraction technologies within standardized omics frameworks, ensuring that technology-associated molecular shifts are interpreted consistently across cultivars, regions and production systems.

### 5.1. Integrated Analytical Approaches

A key future direction is the development of integrated multi-omics pipelines combining metabolomics (NMR, LC–MS/MS, HRMS), physicochemical profiling (pigments, fatty acids, sterols) and microbiome analysis. Microbial communities play a central role in table olive fermentation and in biotransformation pathways of by-products, influencing phenolic stability and metabolite formation. Incorporating metagenomics and metatranscriptomics will allow researchers to link microbial activity with processing-induced molecular changes, particularly in OMWW and pomace valorization streams [2,47].

Such multi-omics integration will provide high-resolution mapping of molecular responses to PEF, UAE, MAE and enzymatic treatments, enabling the development of robust authenticity and quality frameworks that reflect both processing effects and cultivar-intrinsic variation.

### 5.2. Climate-Driven Molecular Monitoring

Climate variability increasingly affects phenolic biosynthesis, secoiridoid stability and overall molecular composition in Greek cultivars (Koroneiki, Kolovi, Adramytiani). Longitudinal monitoring that couples targeted and untargeted metabolomics with climatic data is needed to establish climate-resilient authenticity markers. Chemometric modeling can reveal climate-driven molecular trends and support adaptive quality control strategies [5,48].

These efforts will help maintain reliable molecular fingerprints under changing environmental conditions and ensure that authenticity frameworks remain robust despite climate-induced variability.

### 5.3. National Databases and Harmonization

The development of an interoperable Greek molecular marker database for EVOO, table olives and olive by-products is a strategic priority. The harmonization of analytical protocols between Greek institutions will ensure comparability and reproducibility of the molecular data. Integration with European databases will enhance cross-border authentication, traceability and regulatory alignment [1,2,49].

Such a database will serve as a national reference on processing-induced molecular shifts, cultivar differentiation and climate-responsive marker stability, supporting both scientific research and regulatory decision-making.

### 5.4. Cross-Institutional Collaborations

Progress will require coordinated pilot-plant trials of PEF, UAE, MAE and enzymatic treatments combined with integrated omics readouts. Testing Greek EVOOs and table olives (Koroneiki, Kolovi, Adramytiani) under different climatic scenarios will generate high-value datasets for authenticity, quality and sustainability research. Dissemination of these datasets through FoodOmicsGR_RI will support the development of open access molecular marker libraries and reinforce Greece’s position in omics-enabled olive research [1,2,49].

Strengthening collaboration among universities, research centers, regional authorities and industry stakeholders will accelerate the translation of molecular insights into practical guidelines for processing, authenticity assurance and valorization.

## 6. Conclusions

New extraction techniques offer fresh ways to modify the bioactive makeup of olive products, affecting pigment profiles, oxidation markers, phenolic release, and lignan stability. Key molecular classes can be reshaped using methods like pulsed electric fields, microwave-assisted extraction, ultrasound-assisted extraction, and enzymatic treatments in ways that are pertinent to shelf-life performance, antioxidant capacity, and authenticity assessment. At the same time, these modalities enable targeted recovery of high-value phenolics from olive by-products, supporting realistic circular economy strategies rather than idealized biorefinery scenarios.

A central challenge moving forward is ensuring that processing-induced molecular shifts are interpreted within standardized omics baselines. Without harmonized analytical frameworks, technological interventions may obscure cultivar-specific signatures or complicate authenticity verification, particularly for Greek PDO/PGI cultivars. Greece’s strong metabolomics and microbiome infrastructures provide the resolution needed to monitor processing effects across cultivars, regions and climatic conditions, enabling robust interpretation of molecular changes.

Transforming emerging technologies into proven industrial practices will require coordinated national efforts, such as cross-institutional pilot-plant trials, shared molecular databases, and standardized analytical protocols. Such coordinated efforts are essential for translating laboratory-scale promise into industrial feasibility within the Greek milling landscape, which is dominated by small and medium-sized mills. By taking these steps, processing guidelines can be developed more quickly, climate-resilient quality monitoring can be supported, and new value-added products made from olive by-products can be made.

Overall, Greece is well positioned to contribute significantly to omics-enabled innovation, sustainable processing and circular economy applications in the olive sector. The strategic integration of advanced omics frameworks with emerging extraction technologies can strengthen authenticity assurance, enhance product quality and support evidence-based valorization pathways that align with the structural realities of Greek production systems.

## Data Availability

No new data were created or analyzed in this study. Data sharing is not applicable to this article.

## Abbreviations

The following abbreviations are used in this manuscript:

1-AP	1-Acetoxypinoresinol
EVOO	Extra Virgin Olive Oil
FoodOmicsGR_RI	Greek Research Infrastructure for Food Omics
HRMS	High-Resolution Mass Spectrometry
HT	Hydroxytyrosol
LC-MS/MS	Liquid Chromatography–Tandem Mass Spectrometry
MAE	Microwave-Assisted Extraction
NMR	Nuclear Magnetic Resonance
OMWW	Olive Mill Wastewater
PEF	Pulsed Electric Field
STOCSY	Statistical Total Correlation Spectroscopy
UAE	Ultrasound-Assisted Extraction

## References

[1] Theodoridis G., Pechlivanis A., Thomaidis N.S., Spyros A., Georgiou C.A., Albanis T., Skoufos I., Kalogiannis S., Tsangaris G.T., Stasinakis A.S. (2021). FoodOmicsGR_RI: A Consortium for Comprehensive Molecular Characterisation of Food Products. Metabolites.

[2] Beteinakis S., Papachristodoulou A., Kolb P., Rösch P., Schwarzinger S., Mikros E., Halabalaki M. (2023). NMR-Based Metabolite Profiling and the Application of STOCSY Toward the Quality and Authentication Assessment of European EVOOs. Molecules.

[3] Beteinakis S., Papachristodoulou A., Gogou G., Katsikis S., Mikros E., Halabalaki M. (2020). NMR-Based Metabolic Profiling of Edible Olives—Determination of Quality Parameters. Molecules.

[4] Maestrello V., Solovyev P., Bontempo L., Mannina L., Camin F. (2022). Nuclear Magnetic Resonance Spectroscopy in Extra Virgin Olive Oil Authentication. Compr. Rev. Food Sci. Food Saf..

[5] Drakopoulou S., Orfanakis E., Karagiannaki I., Gaitis F., Skoulika S., Papaioannou A., Boukouvalas G., Petropoulos G., Katsoudas V., Kontzedaki R. (2022). Comparative Evaluation of Different Targeted and Untargeted Analytical Approaches to Assess Greek Extra Virgin Olive Oil Quality and Authentication. Molecules.

[6] Zuin V.G., Ramin L.Z. (2018). Green and Sustainable Separation of Natural Products from Agro-Industrial Waste: Challenges, Potentialities, and Perspectives on Emerging Approaches. Top. Curr. Chem..

[7] Ciriminna R., Meneguzzo F., Fidalgo A., Ilharco L.M., Pagliaro M. (2015). Extraction, Benefits and Valorization of Olive Polyphenols. Eur. J. Lipid Sci. Technol..

[8] Xynos N. (2014). Implementation of Green Extraction and Isolation Methodologies for the Recovery of Bioactive Compounds from Olive-Growing Products and By-Products. Ph.D. Thesis.

[9] Gallardo-Fernández M., Gonzalez-Ramirez M., Cerezo A.B., Troncoso A.M., García-Parrilla M.C. (2022). Hydroxytyrosol in Foods: Analysis, Food Sources, EU Dietary Intake, and Potential Uses. Foods.

[10] Wijaya G.Y.A., Cuffaro D., Bertini S., Digiacomo M., Macchia M. (2024). 1-Acetoxypinoresinol, a Lignan from Olives: Insight into Its Characterization, Identification, and Nutraceutical Properties. Nutrients.

[11] Marakis G., Gaitis F., Mila S., Papadimitriou D., Tsigarida E., Mousia Z., Karpouza A., Magriplis E., Zampelas A. (2021). Attitudes Towards Olive Oil Usage, Domestic Storage, and Knowledge of Quality: A Consumers’ Survey in Greece. Nutrients.

[12] Kaloteraki C., Bousdouni P., Almpounioti K., Ouzaid C., Papagianni O., Sfikti F., Dimitsa E., Tsami D., Sarivasilleiou A.G., Karantonis H.C. (2023). Fortification of Olive Oil with Herbs and Waste by-Products Towards Sustainable Development: Total Antioxidant Capacity, Phenolic Content, and in Vitro Predicted Bioavailability. Appl. Sci..

[13] Montoro-Alonso S., Expósito-Almellón X., Martínez-Baena D., Martínez-Martí J., Rueda-Robles A., Pérez-Gálvez R., Quirantes-Piné R., Lozano-Sánchez J. (2025). Bioactive Enrichment and Sustainable Processing of Vegetable Oils: New Frontiers in Agri-Food Technology. Foods.

[14] Athanasiadis V., Chatzimitakos T., Kotsou K., Kalompatsios D., Bozinou E., Lalas S.I. (2023). Polyphenol Extraction from Food (by) Products by Pulsed Electric Field: A Review. Int. J. Mol. Sci..

[15] Chatzimitakos T., Athanasiadis V., Kalompatsios D., Mantiniotou M., Bozinou E., Lalas S.I. (2023). Pulsed Electric Field Applications for the Extraction of Bioactive Compounds from Food Waste and By-Products: A Critical Review. Biomass.

[16] Clodoveo M.L. (2019). Industrial Ultrasound Applications in the Extra-Virgin Olive Oil Extraction Process: History, Approaches, and Key Questions. Foods.

[17] Clodoveo M.L., Crupi P., Corbo F. (2021). Olive Sound: A Sustainable Radical Innovation. Processes.

[18] Amirante R., Clodoveo M.L. (2016). Developments in the Design and Construction of Continuous Full-scale Ultrasonic Devices for the EVOO Industry. Eur. J. Lipid Sci. Technol..

[19] Zaky A.A., Witrowa-Rajchert D., Nowacka M. (2025). Revolution of Bioactive Compound Extraction: Impacts on Food Safety, Health, and Sustainability. Food Saf. Health.

[20] Sakalasuriya D.D., Jayathilaka N., Seneviratne K.N. (2026). Adulteration of Edible Oils: Additives and Detection Methods—A Systematic Review. J. Am. Oil Chem. Soc..

[21] Emwas A.-H.M., Al-Rifai N., Szczepski K., Alsuhaymi S., Rayyan S., Almahasheer H., Jaremko M., Brennan L., Lachowicz J.I. (2021). You Are What You Eat: Application of Metabolomics Approaches to Advance Nutrition Research. Foods.

[22] Zahran H.A., Fayez S., Zayed A., Azab M.A., Fayek N.M., Zhang L., Capanoglu E., Farag M.A. (2026). Integrated Metabolomic Profiling of Olive Oil, Olive Mill Wastewater, and Pomace from Egyptian Cultivars Based on UHPLC-MS/MS and NMR Coupled with Chemometrics. J. Adv. Res..

[23] Beteinakis S., Papachristodoulou A., Mikros E., Halabalaki M. (2021). from Sample Preparation to NMR-based Metabolic Profiling in Food Commodities: The Case of Table Olives. Phytochem. Anal..

[24] Clodoveo M.L., Moramarco V., Paduano A., Sacchi R., Di Palmo T., Crupi P., Corbo F., Pesce V., Distaso E., Tamburrano P. (2017). Engineering Design and Prototype Development of a Full Scale Ultrasound System for Virgin Olive Oil by Means of Numerical and Experimental Analysis. Ultrason. Sonochem..

[25] Bejaoui M.A., Beltrán G., Sánchez-Ortíz A., Sánchez S., Jiménez A. (2015). Continuous High Power Ultrasound Treatment Before Malaxation, a Laboratory Scale Approach: Effect on Virgin Olive Oil Quality Criteria and Yield. Eur. J. Lipid Sci. Technol..

[26] Attard K., Lia F. (2024). The Antioxidant and Bioactive Potential of Olive Mill Waste. The Power of Antioxidants—Unleashing Nature’s Defense Against Oxidative Stress.

[27] Boskou D., Clodoveo M.L. (2021). Olive Oil.

[28] Clodoveo M.L., Durante V., La Notte D., Punzi R., Gambacorta G. (2013). Ultrasound-assisted Extraction of Virgin Olive Oil to Improve the Process Efficiency. Eur. J. Lipid Sci. Technol..

[29] De Santis S., Cariello M., Piccinin E., Sabbà C., Moschetta A. (2019). Extra Virgin Olive Oil: Lesson from Nutrigenomics. Nutrients.

[30] Marx Í.M.G. (2023). Co-Extraction Technique Improves Functional Capacity and Health-Related Benefits of Olive Oils: A Mini Review. Foods.

[31] Markhali F.S. (2021). Effect of Processing on Phenolic Composition of Olive Oil Products and Olive Mill by-Products and Possibilities for Enhancement of Sustainable Processes. Processes.

[32] Frankel E., Bakhouche A., Lozano-Sánchez J., Segura-Carretero A., Fernández-Gutiérrez A. (2013). Literature Review on Production Process to Obtain Extra Virgin Olive Oil Enriched in Bioactive Compounds. Potential Use of Byproducts as Alternative Sources of Polyphenols. J. Agric. Food Chem..

[33] Peres F., Martins L.L., Ferreira-Dias S. (2014). Laboratory-scale Optimization of Olive Oil Extraction: Simultaneous Addition of Enzymes and Microtalc Improves the Yield. Eur. J. Lipid Sci. Technol..

[34] Leone A., Tamborrino A., Esposto S., Berardi A., Servili M. (2022). Investigation on the Effects of a Pulsed Electric Field (PEF) Continuous System Implemented in an Industrial Olive Oil Plant. Foods.

[35] Kalogianni E.P., Georgiou D., Hasanov J.H. (2019). Olive Oil Processing: Current Knowledge, Literature Gaps, and Future Perspectives. J. Am. Oil Chem. Soc..

[36] Cappelli A., Lupori L., Cini E. (2023). Should Extra Virgin Olive Oil Production Change the Approach? A Systematic Review of Challenges and Opportunities to Increase Sustainability, Productivity, and Product Quality. J. Agric. Eng..

[37] Luaces P., Pérez A.G., Sanz C. (2003). Role of Olive Seed in the Biogenesis of Virgin Olive Oil Aroma. J. Agric. Food Chem..

[38] Rodríguez Ó., Bona S., Stäbler A., Rodríguez-Turienzo L. (2022). Ultrasound-Assisted Extraction of Polyphenols from Olive Pomace: Scale Up from Laboratory to Pilot Scenario. Processes.

[39] Won H.J., Choi A. (2026). from Agri-Food Byproducts to High-Value Bioactive Compounds: A Critical Review Linking Green Recovery and Chemical Profiling to Circular Valorization. Molecules.

[40] Leong S.Y., Duque S.M.M., Conde L.A., Khrisanapant P., Oey I. (2024). Addressing the Opportunities of Non-Thermal Food Processing Technologies in the ASEAN Region Context. Int. J. Food Sci. Technol..

[41] Amirante P., Clodoveo M.L., Leone A., Tamborrino A. (2009). Innovazioni Impiantistiche Per La Produzione E Valorizzazione Dell’olio Di Oliva Nel Rispetto Dell’ambiente. Ital. J. Agron..

[42] Campanini M., Fontanella A., Nardi R. (2015). II Ruolo Degli Omega-3 Nel Paziente Pluripatologico Complesso: Dalle Evidenze Alla Pratica Clinica in Medicina Interna. Ital. J. Med..

[43] Gnerre G.E.P., Frediani R., Risicato R., Rondana M., Nardi R. (2019). Alimentazione, Salute E Longevità Nei Pazienti Adulti. Ital. J. Med..

[44] Kerasioti E., Terzopoulou Z., Komini O., Kafantaris I., Makri S., Stagos D., Gerasopoulos K., Anisimov N.Y., Tsatsakis A., Kouretas D. (2017). Tissue Specific Effects of Feeds Supplemented with Grape Pomace or Olive Oil Mill Wastewater on Detoxification Enzymes in Sheep. Toxicol. Rep..

[45] Dermesonlouoglou E., Limnaios A., Bouskou I., Ntzimani A., Tsevdou M., Taoukis P. (2025). Design, Production and Quality Assessment of Antioxidant-Enriched Olive Paste Dips Using Agro-Food by-Products. Molecules.

[46] Roila R., Primavilla S., Ranucci D., Galarini R., Paoletti F., Altissimi C., Valiani A., Branciari R. (2024). The Effects of Encapsulation on the in Vitro Anti-Clostridial Activity of Olive Mill Wastewater Polyphenolic Extracts: A Promising Strategy to Limit Microbial Growth in Food Systems. Molecules.

[47] Tsoungos A., Pemaj V., Slavko A., Kapolos J., Papadelli M., Papadimitriou K. (2023). The Rising Role of Omics and Meta-Omics in Table Olive Research. Foods.

[48] Skodra C., Titeli V.S., Michailidis M., Bazakos C., Ganopoulos I., Molassiotis A., Tanou G. (2021). Olive Fruit Development and Ripening: Break on Through to the “-Omics” Side. Int. J. Mol. Sci..

[49] Calò F., Girelli C.R., Wang S.C., Fanizzi F.P. (2022). Geographical Origin Assessment of Extra Virgin Olive Oil via NMR and MS Combined with Chemometrics as Analytical Approaches. Foods.

